# Associations of gestational weight gain with offspring thinness and obesity: by prepregnancy body mass index

**DOI:** 10.1186/s12978-018-0585-5

**Published:** 2018-09-04

**Authors:** Nianqing Wan, Li Cai, Weiqing Tan, Ting Zhang, Jiewen Yang, Yajun Chen

**Affiliations:** 1 Department of Maternal and Child Health, School of Public Health, Sun Yat-sen University, Guangzhou, 510000 China; 2 Health Promotion Centre for Primary and Secondary Schools of Guangzhou Municipality, Guangzhou, 510000 China

**Keywords:** Gestational weight gain, Maternal prepregnancy BMI, Childhood thinness, Childhood obesity, Epidemiology

## Abstract

**Background:**

Previous studies indicated that excessive gestational weight gain (GWG) was positively associated with offspring obesity. Nevertheless, little is known about the effect of GWG on offspring thinness. This study aimed to assess the association of GWG with childhood weight status across the full range of weight status by prepregnancy body mass index (BMI).

**Methods:**

We used data from a retrospective study of 33,828 Chinese children aged 6–18 years and their mothers. Children’s weight and height were objectively measured. Maternal GWG and other information were collected by using self-reported questionnaires. Multivariate linear regressions and logistic regressions were applied.

**Results:**

Overall, the prevalence of thinness and overweight/obesity in children were 12.9 and 17.3% respectively (*p* < 0.05). Children’s BMI z-score was on average 0.021 higher for every 1-kg greater GWG. For mothers who were underweight or normal weight before pregnancy, excessive GWG was positively associated with offspring overweight/obesity [OR (95% CI): 1.51 (1.21, 1.90) and 1.30 (1.17, 1.45)], whereas inadequate GWG was associated with increased risk of offspring thinness [OR (95% CI): 1.24 (1.05, 1.46) and 1.17 (1.04, 1.32)]. Similar but non-significant associations were found in prepregnancy overweight mothers. Notably, there was a very high prevalence of child overweight/obesity (30.2%) in prepregnancy overweight subgroup regardless of GWG status.

**Conclusions:**

Inadequate GWG was associated with an increased risk of offspring thinness, whereas excessive GWG was associated with an increased risk of offspring overweight and obesity among prepregnancy underweight and normal weight mothers only.

## Plain English summary

Gestation period is considered as a crucial time for growth, development, and physiological changes in children. Excessive gestational weight gain (GWG) was suggested to be positively associated with offspring obesity. Nevertheless, little is known about the effect of GWG on offspring thinness. This study aimed to assess the association of GWG with childhood weight status across the full range of weight status by prepregnancy body mass index (BMI).

The weight and height of 33,828 Chinese children were objectively measured. These children and their mothers were asked to fill in a self-reported questionnaire which included socio-demographic data, maternal prepregnancy weight (kg) and height (cm), as well as mother’s weight (kg) at delivery and so on.

Overall, the prevalence of thinness and overweight/obesity in children were 12.9 and 17.3% respectively. For mothers who were underweight or normal weight before pregnancy, excessive GWG was positively associated with offspring overweight/obesity, whereas inadequate GWG was associated with increased risk of offspring thinness. Similar but non-significant associations were found in prepregnancy overweight mothers. Notably, there was a very high prevalence of child overweight/obesity (30.2%) in prepregnancy overweight subgroup regardless of GWG status. In addition, children’s BMI z-score was on average 0.021 higher for every 1-kg greater GWG in the overall group.

In conclusion, inadequate GWG was associated with an increased risk of offspring thinness, whereas excessive GWG was associated with an increased risk of offspring overweight and obesity among prepregnancy underweight and normal weight mothers only.

## Background

Childhood obesity has been a serious public health problem worldwide [1]. The global prevalence of childhood overweight and obesity increased rapidly, for example in developed countries, from 16.9% of boys in 1980 to 23.8% in 2013 [2]. Previous of evidences have demonstrated that obesity is linked to medical, neurocognitive and psychological abnormalities which impact children’s health and quality of life [3–5]. Despite the rapidly increasing prevalence of obesity, thinness [i.e. low body mass index (BMI) for age] remains a health concern among children in many countries [6–8]. A global survey of 12–18-year-old girls from 40 countries indicated that the prevalence of moderate/severe thinness was 7.6%, especially with higher levels in Asia [9]. It has been suggested that thinness may affect physical growth of children, lower immunity and increase risk of infection [10–12]. Therefore, prevention of childhood thinness should also be taken into account when it comes to the design and implementation of child weight control programs.

Gestation period is considered as a crucial time for growth, development, and physiological changes in children. Previous studies showed that gestational weight gain (GWG) was positively associated with offspring’s birth weight [13] and BMI in childhood [14, 15], adolescence [16, 17], and adulthood [18]. These associations are complex [19, 20], consequently, epidemiologic studies assessing the association between GWG and child weight status are supposed to control for lifestyle and genetics factors between mother and child to reduce bias. Considering the influence of maternal nutritional status before pregnancy, in 2009, the Institute of Medicine (IOM) recommended ranges of BMI-specific weight gain during pregnancy to achieve optimal pregnancy outcomes. However, whether the effect of inappropriate GWG on child weight status also varies in different maternal prepregnancy BMI category is controversial.

Although many of the previously published studies had investigated the relationship of excessive GWG with offspring obesity [21–23], very few reported the long-term impact of inadequate GWG on child weight status in terms of category structure and limited focused on the adolescent population. One longitudinal study consisted of 3600 participants found that excess GWG was associated with an increase in child BMI z-score in early childhood among normal and overweight mothers, whereas low GWG was not associated with child BMI z-score among all pregnancy BMI groups [24]. Furthermore, to the best of our knowledge, no previous study has investigated the association of inadequate GWG with childhood thinness.

Thus, using data from a large population-based, retrospective study, we aimed to examine the association between GWG and weight status across the full range of weight status in offspring aged 6–18 years, and whether these associations differ by mother’s prepregnancy BMI.

## Methods

Data were obtained from a retrospective study, conducted between September 2015 and June 2016 in Guangzhou, a large city in South China. Multistage cluster sampling method was adopted in the recruitment of participants. In the first stage, 30 schools were randomly selected from 12 districts of Guangzhou using probability proportional to size sampling method. In the second stage, all the children in selected schools and their mothers were invited to participate in our survey. A total of 56,096 mother-child pairs participated in our study, 54,395 children completed physical examination and 42,049 children (with 75% response rate) completed the questionnaire with their mothers. In the study, those with missing information about age, gender, BMI, maternal prepregnancy BMI, or GWG were excluded. Lastly, 33,828 mother-child pairs remained in the analyses.

### Study variables and measurements

Children and their mothers were asked to fill in a self-reported questionnaire which included socio-demographic data, maternal prepregnancy weight (kg) and height (cm), as well as mother’s weight (kg) at delivery. Data of children’s birth and feeding situation, personal and family medical history were also collected. Additionally, children’s daily physical activity/sedentary behavior were reported. Trained staff checked the integrity of completed questionnaires upon returned.

#### Prepregnancy BMI

Prepregnancy BMI (kg/m^2^) was calculated by dividing weight (kg) by the square of height (m^2^). We adopted the Chinese standard [25] to categorize maternal prepregnancy weight status as underweight (prepregnancy BMI < 18.5 kg/m^2^), normal weight (prepregnancy BMI 18.5–23.9 kg/m^2^), and overweight (including obesity: prepregnancy BMI ≥ 24.0 kg/ m^2^).

#### Gestational weight gain

GWG (kg) was calculated as the difference between maternal weight before delivery and weight before pregnancy. According to Chinese standard for prepregnancy BMI categories [25] and IOM recommendations [26], adequate GWG was defined as: 12.5–18.0 kg for initially underweight women (< 18.5 kg/m^2^); 11.5–16.0 kg for normal weight women (18.5–23.9 kg/m^2^); 7.0–11.5 kg for overweight women (24.0–27.9 kg/ m^2^); and 5.0–9.0 kg for obese women (≥ 28 kg/m^2^). Inadequate and excessive GWG were defined as below and above these ranges within each prepregnancy BMI category, respectively.

#### Child weight status

Children’s height (cm) and weight (kg) were measured by qualified technicians according to a standardized protocol. Height was measured to the nearest 0.1 cm using a free-standing stadiometer, with each child wearing no shoes. Weight was measured to an accuracy of 0.1 kg using a calibrated digital scales, with each child wearing only underwear and standing at ease. Age- and sex-specific child BMI z-scores were calculated using the WHO’s Growth Reference. Child overweight and obesity were defined based on the age- and gender-specific BMI cutoffs for school-aged children developed by the Working Group on Obesity in China [27]. Thinness was defined by the age- and gender-specific cutoffs of malnutrition for Chinese children [28].

#### Covariates

Mothers provided information about their education level, family income, gestational diabetes mellitus (yes/no), child’s gestational age (i.e., preterm, full-term, or post-term), birth weight (kg), and feeding patterns in the first 6 months (i.e., exclusive breast feeding, mixed feeding, or exclusive artificial feeding). Physical activity was reported by children, with their mothers’ assist if needed. Physical activity of children was estimated using the International Physical Activity Questionnaire-Short Form (IPAQ-SF) [29]. Children were asked to reported the frequency (days) and duration (hours and minutes in each of those days) of each of the following activities during the last 7 days: 1) vigorous-intensity activity; 2) moderate-intensity activity; 3) walking; and 4) sitting.

### Statistical analysis

Data were analyzed using SAS 9.2. Descriptive statistics were conducted for all variables by GWG categories. Continuous variables were presented as mean and standard deviation (SD), and categorical variables were presented as percentage. One-way ANOVA test and Chi-square test were applied to evaluate the differences of continuous and categorical variables, respectively. Multiple linear regression model was used to examine the association between GWG and children’s current BMI or BMI z-score in each prepregnancy BMI subgroup, and multiple logistic regression analyses were performed to assess the association of GWG (inadequate, adequate, or excessive) with children’s thinness and overweight (including obesity), with adjustment for the potential confounders. Adjusted odds ratio (OR) and 95% confidence interval (CI) were calculated from the logistic regression analyses. In addition, we used the Cochran-Armitage test and linear regression to assess trends of maternal prepregnancy BMI and gestational weight gain between 1996 and 2009. *p* value less than 0.05 was considered statistically significant in all analyses.

## Results

Table 1 presents the general characteristics of mothers and children by GWG. Of the 33,828 participating mothers, the proportion of inadequate, adequate, and excessive GWG in mothers was 44.2, 30.9 and 25.0%, respectively. Overall, the mean maternal GWG was 12.9 ± 5.6 kg, and mean prepregnancy BMI was 20.1 ± 2.3 kg/m^2^. Compared with mothers with inadequate GWG, mothers with adequate or excessive GWG had significantly higher maternal education level and family income. Children whose mothers had excessive GWG were younger and were more likely to be boys, to have higher birth weight, and to be artificially fed during the first 6 months (All *P* < 0.0001).

**Table 1:** Characteristics of mothers and children by gestational weight gain

	*N*	GWG categories			Overall	*P* ^*^
		Inadequate	Adequate	Excessive		
Maternal characteristics						
Prepregnancy BMI, kg/m^2^	33,828	20.1 ± 2.1	20.1 ± 2.2	20.2 ± 2.6	20.1 ± 2.3	0.0136
Gestational weight gain, kg	33,828	8.1 ± 2.4	13.7 ± 1.9	20.2 ± 4.0	12.9 ± 5.6	< 0.0001
GDM, %	33,643	1.3	2.0	2.8	1.9	< 0.0001
Education, %	33,044					< 0.0001
Junior high school or below		37.1	23.3	17.5	27.9	
Senior high school or above		27.2	25.6	25.1	26.2	
College or above		35.7	51.1	57.4	45.9	
Family monthly income, %	33,025					< 0.0001
< 8000 RMB		52.1	47.3	43.3	48.4	
8000~ 15,000 RMB		18.4	22.5	24.5	21.2	
≥ 15,000 RMB		9.2	12.3	15.3	11.7	
Refuse to answer		20.3	17.9	16.9	18.7	
Child characteristics						
Boy, %	33,828	47.7	49.6	51.8	49.3	< 0.0001
Age, year	33,828	12.9 ± 3.5	11.7 ± 3.4	11.2 ± 3.3	12.1 ± 3.5	< 0.0001
Feeding pattern in the first 6 months, %	32,826					< 0.0001
Breast feeding		41.7	38.4	36.6	39.4	
Mixed feeding		45.3	46.8	47.3	46.2	
Artificial feeding		13.0	14.8	16.2	14.4	
Birth weight, kg	32,674	3.0 ± 0.5	3.0 ± 0.5	3.1 ± 0.5	3.0 ± 0.5	< 0.0001
Child BMI, kg/m^2^	33,828	18.3 ± 3.5	18.0 ± 3.6	18.2 ± 3.8	18.2 ± 3.6	< 0.0001
MVPA≥60 min	27,182	35.3	37.4	38.5	36.8	< 0.0001

Values are presented as mean ± SD or percentages

*GWG* gestational weight gain, *BMI* body mass index, *GDM* gestational diabetes mellitus, *MVPA* moderate-to-vigorous physical activity

^*^Differences of continuous and categorical variables between gestational weight gain categories were evaluated by using One-way ANOVA and Chi-square test, respectively

As shown in Table 2, the prevalence of overweight and obesity was higher in children of mothers who had excessive GWG (12.7 and 9.9%) than that in those of mothers had adequate (11.0 and 6.8%) or inadequate GWG (8.8 and 5.1%). Whereas the prevalence of offspring thinness was higher in the inadequate GWG group (14.4%) than that in adequate (12.6%) or excessive GWG group (10.6%). This trend also existed within each prepregnancy BMI subgroup. Mothers with prepregnancy overweight, regardless of GWG category, were more likely to have overweight or obese offspring and less likely to have normal weight offspring compared with those with prepregnancy normal weight or underweight. In particular, among the prepregnancy overweight group, there was very high combined prevalence of offspring overweight and obesity in both the adequate (around 31%) as well as the excessive GWG group (around 36%).

**Table 2:** Children’s weight status by GWG stratified by prepregnancy BMI categories

GWG categories	Children weight status (%)				*P* ^*^
	Thinness	Normal	Overweight	Obesity	
Overall	12.9	69.8	10.5	6.8	< 0.0001
Inadequate	14.4	71.6	8.8	5.1	
Adequate	12.6	69.7	11.0	6.8	
Excessive	10.6	66.7	12.7	9.9	
Prepregnancy underweight	19.0	69.3	7.4	4.3	< 0.0001
Inadequate	21.0	68.5	7.2	3.4	
Adequate	18.0	71.0	6.9	4.1	
Excessive	16.6	68.5	8.6	6.3	
Prepregnancy normal weight	11.4	70.5	11.0	7.1	< 0.0001
Inadequate	12.6	72.6	9.3	5.5	
Adequate	11.3	69.9	11.8	6.9	
Excessive	9.4	67.1	13.4	10.1	
Prepregnancy overweight	6.5	63.3	15.8	14.4	< 0.0001
Inadequate	8.0	72.4	11.0	8.6	
Adequate	6.5	62.4	16.7	14.3	
Excessive	5.4	58.2	18.2	18.2	

Values are presented as percentages

*GWG* gestational weight gain

^*^Differences of child weight status between gestational weight gain categories were evaluated by using Chi-square test

As a continuous variable, GWG was positively associated with children’s mean BMI after adjustment for all confounding factors, though the effect size was small (0.049 kg/m^2^ change in children’s mean BMI per 1-kg change in GWG). After stratified by prepregnancy BMI categories, the association remained statistically significant only in the prepregnancy underweight and normal weight subgroup. Similar associations were also observed between GWG and offspring BMI z-score (Table 3).

**Table 3:** Association between GWG and children’s BMI stratified by prepregnancy BMI categories

GWG, kg	Children’s BMI, kg/m^2^			Children’s BMI z-score		
	Coefficient	Standardized coefficient	*P*	Coefficient	Standardized coefficient	*P*
Overall	0.049	0.075	< 0.0001	0.021	0.090	< 0.0001
Prepregnancy underweight	0.037	0.063	< 0.0001	0.017	0.078	< 0.0001
Prepregnancy normal weight	0.058	0.088	< 0.0001	0.025	0.105	< 0.0001
Prepregnancy overweight	0.024	0.034	0.2096	0.008	0.033	0.2429

Adjusted for child’s age, gender, school, gestational age, birth weight, feeding patterns in the first 6 months, child’s physical activities, maternal prepregnancy BMI, maternal education level, family monthly income and gestational diabetes mellitus

*GWG* gestational weight gain, *BMI* body mass index

In logistic regression models, after adjustment for confounders, excessive GWG was associated with increased risk of offspring overweight/obesity [OR (95% CI): 1.31 (1.20, 1.43)] and lower risk of thinness [OR (95% CI): 0.80 (0.72, 0.90)] compared with adequate GWG (Table 4). In contrast, inadequate GWG was associated with elevated risk of offspring thinness [OR (95% CI): 1.18 (1.08, 1.30)] and lower odds of overweight/obesity [OR (95% CI): 0.86 (0.79, 0.94)] compared with adequate GWG. After stratified by prepregnancy BMI categories, we found positive associations between excessive GWG and offspring overweight/obesity, as well as between inadequate GWG and childhood thinness only among prepregnancy underweight and normal weight mothers. There was also a negative relation of inadequate GWG and risk of offspring overweight/obesity in prepregnancy normal weight and overweight/obese mothers.

**Table 4:** Association between GWG and child weight status stratified by prepregnancy BMI categories

GWG categories	Childhood thinness		Childhood overweight/obesity	
	OR (95% CI)	*P*	OR (95% CI)	*P*
Overall				
Inadequate	1.18(1.08, 1.30)	0.0004	0.86(0.79, 0.94)	0.0009
Adequate	1.00		1.00	
Excessive	0.80(0.72, 0.90)	0.0002	1.31(1.20, 1.43)	< 0.0001
Prepregnancy underweight				
Inadequate	1.24(1.05, 1.46)	0.01	1.19(0.96, 1.47)	0.1127
Adequate	1.00		1.00	
Excessive	0.91(0.74, 1.11)	0.3458	1.51(1.21, 1.90)	0.0004
Prepregnancy normal weight				
Inadequate	1.17(1.04, 1.32)	0.0071	0.80(0.72, 0.89)	< 0.0001
Adequate	1.00		1.00	
Excessive	0.76(0.66, 0.88)	0.0002	1.30(1.17, 1.45)	< 0.0001
Prepregnancy overweight				
Inadequate	1.20(0.69, 2.09)	0.5243	0.55(0.39, 0.79)	0.0013
Adequate	1.00		1.00	
Excessive	0.84(0.48, 1.47)	0.5363	1.16(0.87, 1.54)	0.3214

Adjusted for child’s age, gender, school, gestational age, birth weight, feeding patterns in the first 6 months, child’s physical activities, maternal prepregnancy BMI, maternal education level, family monthly income and gestational diabetes mellitus

*GWG* gestational weight gain, *BMI* body mass index, *OR* odds ratio, *CI* confidence interval

**Fig. 1:**
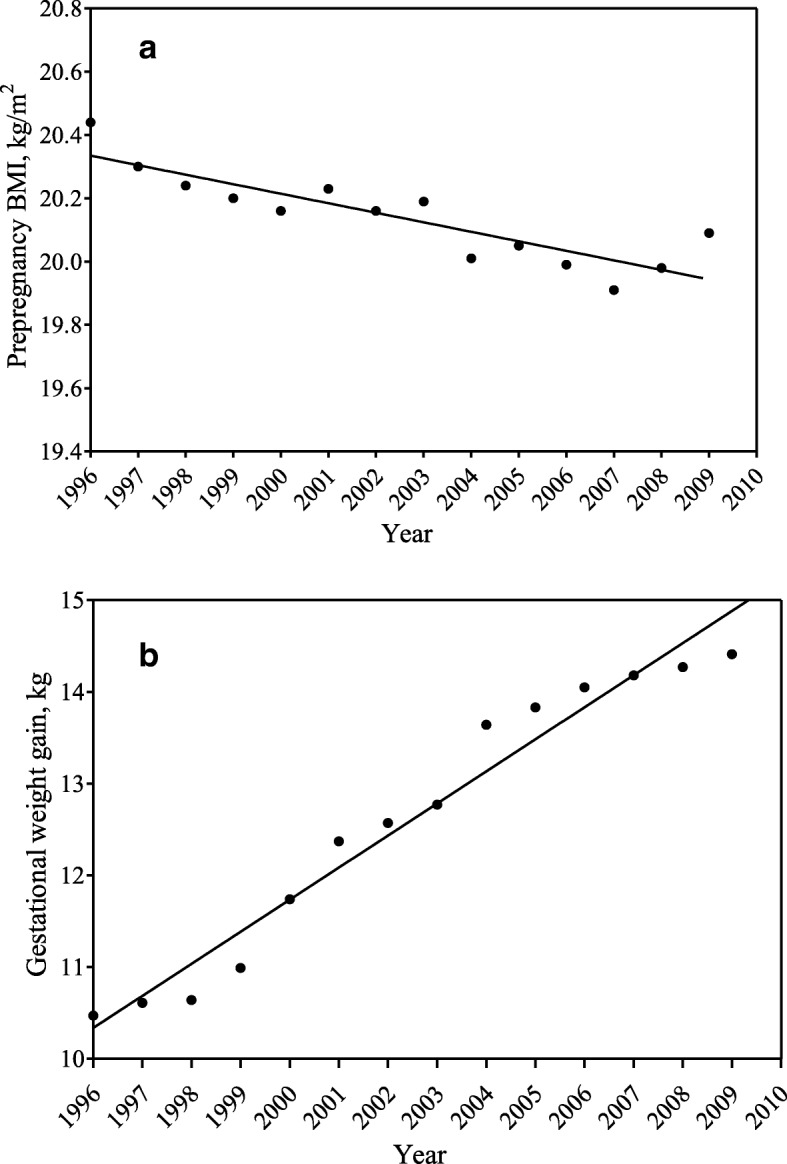
**a** Trend of the mean maternal prepregnancy BMI during 1996–2009. **b** Trend of the mean maternal gestational weight gain during 1996–2009

Table 5 presents the trend of maternal prepregnancy BMI and GWG between 1996 and 2009. The average prepregnancy BMI decreased slightly from 20.4 kg/m^2^ in 1996 to 20.1 kg/m^2^ in 2009 (*P* < 0.001) (Fig. 1a). Accordingly, the prevalence of prepregnancy underweight increased from 22.7 to 24.8% (*P* < 0.0001). Especially noteworthy is that the average GWG increased significantly from 10.5 kg to 14.4 kg (*P* < 0.001) (Fig. 1b), with the prevalence of excessive GWG rising from 16.2 to 31.8%. Meanwhile, the prevalence of inadequate GWG decreased from 58.7 to 33.4% between 1996 and 2009. The increasing trend of GWG remained significant within each prepregnancy BMI category.

**Table 5:** Trends for maternal prepregnancy BMI and gestational weight gain during 1996 ~ 2009

	1996~ 2000	2001~ 2005	2006~ 2009	Overall	P^*^ for trend
Prepregnancy BMI categories^§^					
Underweight	22.7	23.1	24.8	23.5	0.0001
Normal weight	71.0	71.4	70.1	70.8	0.0784
Overweight	6.3	5.6	5.0	5.6	< 0.0001
GWG categories					
Overall^#^					
Inadequate	58.7	42.1	33.4	44.2	< 0.0001
Adequate	25.2	31.9	34.7	30.9	< 0.0001
Excessive	16.2	26.0	31.8	25.0	< 0.0001
Prepregnancy underweight^#^					
Inadequate	58.6	42.6	34.9	44.5	< 0.0001
Adequate	24.9	32.3	37.1	31.8	< 0.0001
Excessive	16.6	25.1	28.0	23.7	< 0.0001
Prepregnancy normal weight^#^					
Inadequate	61.0	43.2	34.2	45.6	< 0.0001
Adequate	23.8	31.5	33.9	30.0	< 0.0001
Excessive	15.2	25.3	31.9	24.5	< 0.0001
Prepregnancy overweight^#^					
Inadequate	32.4	25.9	15.1	24.9	< 0.0001
Adequate	42.0	36.3	35.1	37.8	0.0069
Excessive	25.6	37.8	49.8	37.2	< 0.0001

Values are presented as percentages

*GWG* gestational weight gain, *BMI* body mass index

^*^The Cochran-Armitage trend test were used for comparing the prevalence of each prepregnancy BMI and GWG category in different years

^#^Significant proportions (*P* < 0.0001) of GWG category during different period

^§^Significant proportions (*P* < 0.0001) of prepregnancy BMI category during different period

## Discussion

In this study, heterogeneity of relationship between GWG and offspring weight status stratified by prepregnancy BMI categories was observed. For mothers with prepregnancy underweight or normal weight, inadequate GWG increased the risk of thinness, while excessive GWG increased the risk of overweight/obesity in children aged 6–18 years, compared with mothers with adequate GWG. However, such associations were not found in children whose mothers were overweight/obese before pregnancy.

The positive overall association between excessive GWG and increased risk of children overweight/obesity in our study was consistent with previous studies [30–32]. Nevertheless, results from different studies on the prepregnancy BMI-specific associations between excess GWG and child weight status have been inconsistent. Hinkle et al. [24] found a positive association between excess GWG and child BMI Z-score among normal and overweight mothers only, whereas Wrotniak et al. [33] found that the impact of excess GWG on offspring overweight was greatest in prepregnancy underweight women. Notably, we observed such association only among mothers who were underweight or normal weight, but not among overweight/obesity mothers. This result may reflect that there are many factors contributing to obesity among children born to overweight or obese mothers. These children may have already at increased risk of overweight/obesity because of genetic predisposition or familial lifestyle factors [20]. Indeed, we observed that both the prevalence of overweight and obesity were alarmingly high in children born to mothers with prepregnancy overweight, regardless of GWG status. A systematic review also lends support that prepregnancy overweight was associated with an increased risk of offspring overweight/obesity, compared with mothers with normal weight [34]. Therefore, in addition to preventing excess GWG, these findings further highlight the importance of avoiding prepregnancy overweight for the prevention of childhood overweight/obesity.

Research evaluating the relationship between inadequate GWG and childhood overweight/obesity has produced mixed conclusions. Two studies [30, 35] found that low GWG was associated with an increase in obesity risk, whereas another study [36] found no association between low GWG and child obesity in any prepregnancy BMI group. In contrast, we found a negative association between inadequate GWG and child overweight/obesity among prepregnancy non-underweight mothers. Our findings are intriguing for further studies to explore the reasons for such variation in different populations.

Our work also extends previous studies in which it examined the effect of maternal GWG on offspring thinness and found that inadequate GWG increased the risk of offspring thinness among mothers who were underweight or normal weight before pregnancy. Sarah et al. [37] found that low GWG during the first trimester of pregnancy was associated with reduced child BMI at age 4.5 years, independent of prepregnancy BMI and birth size, which provided some support for our findings. This suggests the need to promote adequate GWG for the prevention of not only childhood overweight/obesity but also thinness.

Interestingly, we found a negative association between excess GWG and offspring thinness, especially among normal weight mothers. This should be interpreted with caution and future studies are warranted to confirm the present finding. In the other hand, when considering the genetic variation among populations, the appropriate GWG may vary by ethnic group. The IOM category has been recommended by the Chinese Society of Nutrition and was applied in our study. However, the IOM recommended levels of pregnancy weight gain might not be optimal for Chinese women, especially for those with prepregnancy overweight women (BMI ≥ 24 kg/m^2^). Hence, further studies are required to access the applicability of IOM guidelines and to investigate whether new GWG classification guidelines are needed in China.

There are several important potential pathways by which GWG may relate to offspring BMI later in life. GWG is suggested to exert an indirect effect on child’s weight status through birth weight [24]. However, despite the positive association of birth weight with later BMI [16], we found that the associations between GWG and child weight status remained fairly robust after adjusted for child’s birth weight and other confounders.

Furthermore, measuring maternal nutrition or GWG is recognized as a proxy of the fetal nutrition environment [37]. Changes in fetal nutrition status or endocrine system may induce lasting alterations in physiology, metabolism, or organ structure of individuals, predisposing them to later metabolic disorders [38, 39]. Therefore, inadequate or excessive GWG may lead to partially nutritional modification of intrauterine exposures of the fetus. Firstly, intrauterine exposures may have sustained influence on offspring weight by determining fetus’s body composition and programming of hypothalamic appetite regulation centers [40, 41]. Secondly, maternal over-nutrition may overstimulate fetal pancreatic Beta-cells and accordingly bring about fetal hyper-insulinism, which may have permanent effects on offspring pancreatic structure and function, resulting in impaired glucose tolerance and obesity in adolescence [40, 42]. Thirdly, central leptin sensitivity in offspring may also be affected by prenatal over-nutrition [43]. Changes in leptin levels during key periods of hypothalamic development may induce long-term and potentially irreversible effects on metabolism in later life [41]. It is not likely to test these possibilities based on this study, but these findings support that intrauterine exposures may mediate the relations of GWG and offspring weight status. Thus, promoting measures to ensure appropriate GWG is important for the prevention of childhood thinness and obesity regardless of maternal prepregnancy BMI.

Although achieving adequate GWG plays an important role in the long-term health of offspring, the average maternal GWG and the prevalence of excessive GWG were significantly increased during 1996 to 2009. It is noteworthy that the prevalence of excessive GWG during 2006–2009 was approximately twice as high as during 1996–2000. In contrast, both the percentage of women gaining above IOM recommendations and mean GWG in America only slightly increased from 2000 through 2009 [44]. The rapid economic development, improvement of food environment, as well as low level of physical activity may account for the increasing prevalence of excessive GWG women in China. Nevertheless, other reasons for this trend warrant further investigation. These trends suggest that it is important for policymakers and health professionals to develop practical strategies for healthy weight management during pregnancy.

There are several strengths of this study. Firstly, we related GWG to the risk of offspring thinness in childhood and adolescence. Our findings not only support other evidences suggesting to avoid excess weight gain during pregnancy but also emphasis the negative impact of inadequate GWG. Secondly, we considered the broad age spectrum of children (6–18 years). Finally, this is the first study to report the recent secular trends in prepregnancy BMI and maternal GWG among Chinese women. A rapidly increasing trend in GWG was identified and warranted more attention from researchers and clinicians.

A major limitation in our study is that the prepregnancy BMI and maternal GWG were assessed based on self-reported data rather than objective measurement, which may result in recall bias. However, previous studies found that self-reported weight and height were generally valid for identifying associations in epidemiological studies [45, 46]. Besides, a potential selection bias can’t be excluded in this study, because children who didn’t respond to questionnaire survey were more likely to be boys (55.4% vs. 49.9%) and be older (13.24 year vs. 12.32 year) than those who completed the questionnaire. To account for this, we additionally carried out subgroup analyses by gender and age. Results show that the associations between inappropriate GWG and offspring weight status were similar in different age group, and were slightly stronger in boys than in girls. Therefore, the associations we report here are possible to be underestimated, but not overestimated.

## Conclusions

Our study suggested that inadequate GWG was associated with an increased risk of offspring thinness at 6–18 years of age, whereas excessive GWG was associated with an increased risk of offspring obesity. The adverse effects of inadequate or excessive GWG on offspring weight status were only found for initially underweight and normal-weight mothers, but not for overweight/obese mothers. More attention should be paid to the influences of maternal weight change during pregnancy on childhood weight status to prevent the inter-generational cycle of thinness and obesity.

## Abbreviations

BMI: Body mass index
CI: Confidence interval
GDM: Gestational diabetes mellitus
GWG: Gestational weight gain
IOM: the Institute of Medicine
MVPA: Moderate-to-vigorous physical activity
OR: Odds ratio

## References

[1] Han JC, Lawlor DA, Kimm SY (2010). Childhood obesity. Lancet.

[2] Ng M, Fleming T, Robinson M, Thomson B, Graetz N, Margono C, Mullany EC, Biryukov S, Abbafati C, Abera SF (2014). Global, regional, and national prevalence of overweight and obesity in children and adults during 1980-2013: a systematic analysis for the Global Burden of Disease Study 2013. Lancet.

[3] Pulgaron ER (2013). Childhood obesity: a review of increased risk for physical and psychological comorbidities. Clin Ther.

[4] Owens S, Galloway R (2014). Childhood obesity and the metabolic syndrome. Curr Atheroscler Rep.

[5] Sweat V, Yates KF, Migliaccio R, Convit A (2017). Obese adolescents show reduced cognitive processing speed compared with healthy weight peers. Child Obes.

[6] Noh JW, Kim YE, Park J, Oh IH, Kwon YD (2014). Impact of parental socioeconomic status on childhood and adolescent overweight and underweight in Korea. J Epidemiol.

[7] Martinez-Vizcaino V, Solera MM, Notario PB, Sanchez LM, Garcia-Prieto JC, Torrijos NC, Arias PN, Salcedo AF, Rodriguez-Artalejo F (2012). Trends in excess of weight, underweight and adiposity among Spanish children from 2004 to 2010: the Cuenca study. Public Health Nutr.

[8] Lee G, Ham OK (2015). Factors affecting underweight and obesity among elementary school children in South Korea. Asian Nurs Res.

[9] Candler T, Costa S, Heys M, Costello A, Viner RM (2017). Prevalence of thinness in adolescent girls in low- and middle-income countries and associations with wealth, food security, and inequality. J Adolesc Health.

[10] Wolnicka K, Jarosz M, Jaczewska-Schuetz J, Taraszewska AM (2016). Differences in the prevalence of overweight, obesity and underweight among children from primary schools in rural and urban areas. Ann Agric Environ Med.

[11] Schaible UE, Kaufmann SH (2007). Malnutrition and infection: complex mechanisms and global impacts. PLoS Med.

[12] Gaayeb L, Sarr JB, Cames C, Pincon C, Hanon JB, Ndiath MO, Seck M, Herbert F, Sagna AB, Schacht AM (2014). Effects of malnutrition on children’s immunity to bacterial antigens in northern Senegal. Am J Trop Med Hyg.

[13] Goldstein RF, Abell SK, Ranasinha S, Misso M, Boyle JA, Black MH, Li N, Hu G, Corrado F, Rode L (2017). Association of gestational weight gain with maternal and infant outcomes: a systematic review and meta-analysis. JAMA.

[14] Guo L, Liu J, Ye R, Liu J, Zhuang Z, Ren A (2015). Gestational weight gain and overweight in children aged 3-6 years. J Epidemiol.

[15] Leonard SA, Petito LC, Rehkopf DH, Ritchie LD, Abrams B (2017). Weight gain in pregnancy and child weight status from birth to adulthood in the United States. Pediatr Obes.

[16] Oken E, Rifas-Shiman SL, Field AE, Frazier AL, Gillman MW (2008). Maternal gestational weight gain and offspring weight in adolescence. Obstet Gynecol.

[17] Laitinen J, Jaaskelainen A, Hartikainen AL, Sovio U, Vaarasmaki M, Pouta A, Kaakinen M, Jarvelin MR (2012). Maternal weight gain during the first half of pregnancy and offspring obesity at 16 years: a prospective cohort study. BJOG.

[18] Mamun AA, O’Callaghan M, Callaway L, Williams G, Najman J, Lawlor DA (2009). Associations of gestational weight gain with offspring body mass index and blood pressure at 21 years of age: evidence from a birth cohort study. Circulation.

[19] Lawlor DA, Lichtenstein P, Fraser A, Langstrom N (2011). Does maternal weight gain in pregnancy have long-term effects on offspring adiposity? A sibling study in a prospective cohort of 146,894 men from 136,050 families. Am J Clin Nutr.

[20] Branum AM, Parker JD, Keim SA, Schempf AH (2011). Prepregnancy body mass index and gestational weight gain in relation to child body mass index among siblings. Am J Epidemiol.

[21] Mamun AA, Mannan M, Doi SA (2014). Gestational weight gain in relation to offspring obesity over the life course: a systematic review and bias-adjusted meta-analysis. Obes Rev.

[22] Fraser A, Tilling K, Macdonald-Wallis C, Sattar N, Brion MJ, Benfield L, Ness A, Deanfield J, Hingorani A, Nelson SM (2010). Association of maternal weight gain in pregnancy with offspring obesity and metabolic and vascular traits in childhood. Circulation.

[23] Mourtakos SP, Tambalis KD, Panagiotakos DB, Antonogeorgos G, Alexi CD, Georgoulis M, Saade G, Sidossis LS (2017). Association between gestational weight gain and risk of obesity in preadolescence: a longitudinal study (1997–2007) of 5125 children in Greece. J Hum Nutr Diet.

[24] Hinkle SN, Sharma AJ, Swan DW, Schieve LA, Ramakrishnan U, Stein AD (2012). Excess gestational weight gain is associated with child adiposity among mothers with normal and overweight prepregnancy weight status. J Nutr.

[25] Chen C, Lu FC (2004). The guidelines for prevention and control of overweight and obesity in Chinese adults. Biomed Environ Sci.

[26] to IOMU, Guidelines RIPW (2009). Weight gain during pregnancy: reexamining the guidelines.

[27] Group of China Obesity Task Force (2004). Body mass index reference norm for screening overweight and obesity in Chinese children and adolescents. Zhonghua Liu Xing Bing Xue Za Zhi.

[28] China NHAF (2014). Screening standard for malnutrition of school-age children and adolescents.

[29] Lee PH, Macfarlane DJ, Lam TH, Stewart SM (2011). Validity of the international physical activity questionnaire short form (IPAQ-SF): a systematic review. Int J Behav Nutr Phys Act.

[30] Stuebe AM, Forman MR, Michels KB (2009). Maternal-recalled gestational weight gain, pre-pregnancy body mass index, and obesity in the daughter. Int J Obes.

[31] Ensenauer R, Chmitorz A, Riedel C, Fenske N, Hauner H, Nennstiel-Ratzel U, von Kries R (2013). Effects of suboptimal or excessive gestational weight gain on childhood overweight and abdominal adiposity: results from a retrospective cohort study. Int J Obes.

[32] Shao T, Tao H, Ni L, Sun Y, Yan S, Gu C, Cao H, Huang K, Hao J, Tao F (2016). Maternal pre-pregnancy body mass index and gestational weight gain with preschool children’s overweight and obesity. Zhonghua Yu Fang Yi Xue Za Zhi.

[33] Wrotniak BH, Shults J, Butts S, Stettler N (2008). Gestational weight gain and risk of overweight in the offspring at age 7 y in a multicenter, multiethnic cohort study. Am J Clin Nutr.

[34] Yu Z, Han S, Zhu J, Sun X, Ji C, Guo X (2013). Pre-pregnancy body mass index in relation to infant birth weight and offspring overweight/obesity: a systematic review and meta-analysis. PLoS One.

[35] Crozier SR, Inskip HM, Godfrey KM, Cooper C, Harvey NC, Cole ZA, Robinson SM (2010). Weight gain in pregnancy and childhood body composition: findings from the Southampton Women’s survey. Am J Clin Nutr.

[36] von Kries R, Ensenauer R, Beyerlein A, Amann-Gassner U, Hauner H, Rosario AS (2011). Gestational weight gain and overweight in children: results from the cross-sectional German KiGGS study. Int J Pediatr Obes.

[37] Wilcox SK. Risk factors for maternal low weight gain during pregnancy and associations with offspring body size and fat distribution. Atlanta: Emory University. Health Sciences, Public Health. 2006. p. 104–7.

[38] Barker DJ (1998). In utero programming of chronic disease. Clin Sci (Lond).

[39] Godfrey KM, Barker DJ (2000). Fetal nutrition and adult disease. Am J Clin Nutr.

[40] Oken E, Gillman MW (2003). Fetal origins of obesity. Obes Res.

[41] Bouret SG (2009). Early life origins of obesity: role of hypothalamic programming. J Pediatr Gastroenterol Nutr.

[42] Silverman BL, Rizzo TA, Cho NH, Metzger BE (1998). Long-term effects of the intrauterine environment. The Northwestern University diabetes in pregnancy center. Diabetes Care.

[43] Ferezou-Viala J, Roy AF, Serougne C, Gripois D, Parquet M, Bailleux V, Gertler A, Delplanque B, Djiane J, Riottot M, Taouis M (2007). Long-term consequences of maternal high-fat feeding on hypothalamic leptin sensitivity and diet-induced obesity in the offspring. Am J Physiol Regul Integr Comp Physiol.

[44] Johnson JL, Farr SL, Dietz PM, Sharma AJ, Barfield WD, Robbins CL (2015). Trends in gestational weight gain: the pregnancy risk assessment monitoring system, 2000-2009. Am J Obstet Gynecol.

[45] Spencer EA, Appleby PN, Davey GK, Key TJ (2002). Validity of self-reported height and weight in 4808 EPIC-Oxford participants. Public Health Nutr.

[46] Stommel M, Schoenborn CA (2009). Accuracy and usefulness of BMI measures based on self-reported weight and height: findings from the NHANES & NHIS 2001-2006. BMC Public Health.

